# Diagnosis, Management, and Associated Comorbidities of Polycystic Ovary Syndrome: A Narrative Review

**DOI:** 10.7759/cureus.58733

**Published:** 2024-04-22

**Authors:** Rutuja Choudhari, Surekha Tayade, Aakriti Tiwari, Prasiddhi Satone

**Affiliations:** 1 Department of Obstetrics and Gynaecology, Datta Meghe Institute of Higher Education and Research, Wardha, IND

**Keywords:** anovulation, reproductive age group, endocrine disorder, infertility, hyperandrogenism, insulin resistance, polycystic ovary syndrome (pcos)

## Abstract

Polycystic ovary syndrome (PCOS) is the most widespread and diverse endocrine health issue affecting many adolescent-aged women globally. It is the most frequent illness in reproductive-aged women. According to the Rotterdam criteria, two out of three elements: oligo-anovulation, hyperandrogenism, and polycystic ovaries (defined as having at least one ovary with an ovarian volume > 10 mL and/or 12 or more follicles measuring 2 to 9 mm in diameter) are present in PCOS. Conducted studies show epigenetics, environmental toxins, stress, and food as external factors as well as inflammation, oxidative stress, hyperandrogenism, insulin resistance, and obesity as internal factors related to PCOS. Although a portion of the mechanism associated with the occurrence of PCOS has been identified, there is still much to learn about the exact etiology and pathophysiology. The main debate covers the best ways to diagnose and treat this disease in adolescents. Early detection is crucial because of the disease's long-term effects on metabolic and reproductive health. Before beginning treatment for this group of young women, a firm diagnosis may not be made. Various criteria are used to diagnose PCOS patients. A person with PCOS has a chance of developing several comorbidities and health effects. PCOS patients are at risk of cardiac diseases, metabolic syndromes, resistance to insulin, infertility, and many more. There are numerous medications available for PCOS therapy that need a methodical approach. However, changing one's lifestyle should come first. There is proof in the support of the usage of several medications for PCOS, including mucolytic agents, Hydroxymethylglutaryl-CoA (HMG-CoA) reductase inhibitors, gliptins (oral diabetic medication), glucose-like peptide-1 receptor analogues, glitazones, and sodium-glucose cotransporter protein-2 (SGLT2) inhibitors. A comprehensive, systematic, schematic therapy approach is crucial for the treatment of PCOS.

## Introduction and background

Polycystic ovary syndrome (PCOS) is a highly prevalent endocrine condition that affects women in the reproductive age group globally [1]. High levels of androgen hormone, impaired insulin sensitivity, oversized and malfunctioning ovaries, and other variables are usually correlated with this condition [2]. Before menopause, estimates show that 1/10 women struggle with PCOS and related issues [3]. There is still much to learn about the exact etiology and pathophysiology PCOS [4,5]. There is evidence pointing toward the association of various factors, including insulin resistance (IR), epigenetics, environmental factors, hyperandrogenism, and genetics. The risk of other consequences such as type 2 diabetes mellitus [5,6], cardiovascular diseases [5,6], metabolic syndrome [6], and anxiety and depression [7] is also something that should be mentioned. Pathognomonic symptoms for PCOS in adults typically appear throughout adolescence. The detection of PCOS in its early stages is important for effectively managing its chronic metabolic and reproductive health effects [8]. When this long-term health issue is found in adolescent women, treatment should be personalized and consider the situation's peculiarities.

## Review

Methodology 

**Figure 1:**
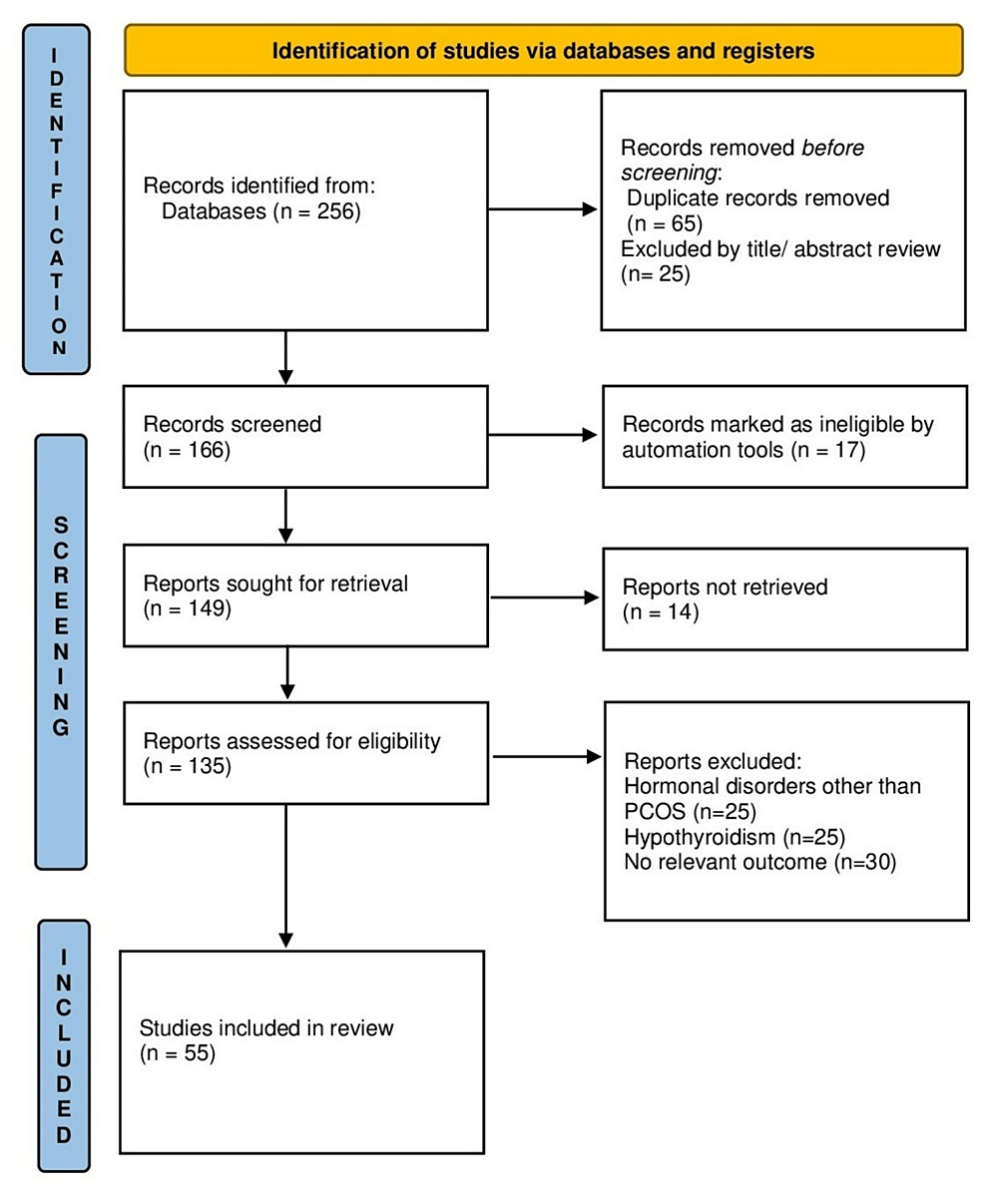
PRISMA diagram PCOS: polycystic ovary syndrome; PRISMA: preferred reporting items for systematic reviews and meta-analyses

The search methodology followed an approach for the identification of relevant studies for the review. The process involves searching for various screening articles, defining inclusion, databases, and exclusion criteria, and selecting the final approach for the review. The search covered papers published from the databases' inception without explicit date constraints. The search strategy incorporated a combination of key terms and medical subject heading terms related to PCOS, diagnosis, comorbidities, and management strategies. The key terms that were used were PCOS, insulin resistance, hyperandrogenism, anovulation, and infertility. Case reports and editorials were excluded from the review. The initial screening involves reviewing the titles and abstracts of the identified articles based on the inclusion and exclusion criteria. A total of 55 articles met the inclusion criteria and were included in the final review. The following preferred reporting items for systematic reviews and meta-analyses (PRISMA) flow diagram (Figure 1) provides a visual representation of the search methodology, showing the number of articles identified, screened, and included in the final review.

Diagnosis of PCOS

**Figure 2:**
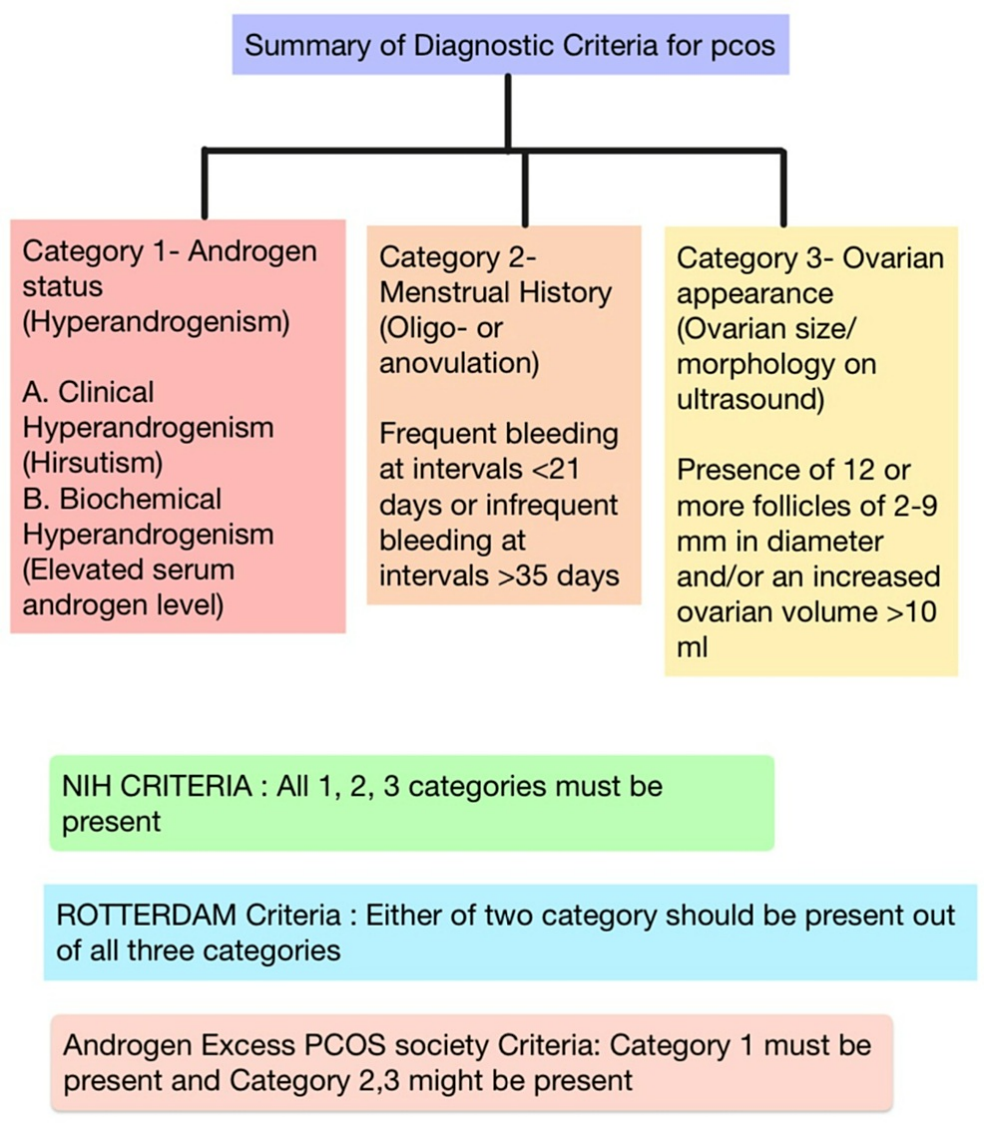
Summary of diagnostic criteria for PCOS PCOS: polycystic ovary syndrome; NIH: National Institutes of Health Figure created by the authors.

As PCOS is the most frequent endocrine illness observed in adolescent age group women, its diagnosis and further proper treatment are equally important. The main discussion surrounds the diagnosis and management of PCOS. Milestones of management include the early diagnosis of PCOS. There are various criteria used for the diagnosis of this illness. Criteria include National Institutes of Health criteria, Rotterdam criteria, androgen excess PCOS society criteria [9]. Mainly clinical diagnosis depends upon irregular menstrual cycle, excess facial hair growth, gain of weight, acne, and infertility [9]. Radiological diagnosis is also a mainstay which includes multiple cysts in ovaries. For proper management of PCOS, a systematic approach toward the treatment of clinical features and related comorbidities is important. Figure 2 summarizes the diagnostic criteria for PCOS [10].

Evaluation of PCOS patients and associated morbidities

Physical examination includes observing different signs of PCOS, which include cutaneous signs such as androgenic alopecia, acne, acanthosis nigricans, skin tags, terminal hair development, and male pattern baldness in women [11]. Other signs include gain in muscular mass, deepening of the voice, or development of clitoromegaly. Moreover, it is possible to have high levels of virilizing androgens, severe insulin resistance, or hidden ovarian or adrenal tumors [12]. Notably, acanthosis nigricans and skin tags are usually seen in overweight, insulin-resistant women suffering from PCOS [13].

In a comprehensive investigation of individuals with clinical hyperandrogenism, PCOS was identified in 72.1% of the 950 participants [14]. The modified Ferriman-Gallwey score [10,15] remains the approach used most frequently to visually evaluate hirsutism. A few studies have shown a connection between metabolic syndrome [16], insulin resistance [17], and androgenic alopecia. Compared to hirsutism, other studies revealed that androgenic alopecia and acne are poor indicators of hyperandrogenism in PCOS [18].

One of Stein et al.’s primary PCOS symptoms was infertility, which is a frequent presenting issue today [19,20]. Primary infertility was reported in nearly 50% of PCOS-affected women in a large sample, and secondary infertility was reported in 25% [21]. Longer anovulation intervals are probably linked to higher infertility. According to estimates, 70-90% of abnormalities in ovaries are typically seen in PCOS patients and are the most common reason for ovulatory dysfunction [22]. Although it is believed that oligo- or anovulation is the primary cause of infertility, there are additional possible causes such as decreased oocyte competence [23,24] and endometrial alterations that prevent implantation [24]. Subfertility and delayed conception have also been linked to PCOS [25].

The relationships show proof of fetal programming of adult illnesses between intrauterine growth restriction and higher risks of type 2 diabetes, ischemic heart disease, and hypertension [26]. A minority of girls who are born tiny for gestational age also run the risk of experiencing early adrenarche, IR, or PCOS. Limited evidence suggests that in some groups, intrauterine growth restriction may be linked to the later emergence of PCOS [27].

The evidence is in favor of the idea that metabolic abnormalities and PCOS symptoms can be aggravated by postpartum obesity and fast weight gain [28]. In addition to having greater rates of hirsutism and oligomenorrhea, endometrial cancer in young women increases the likelihood of infertility and nulliparity [29]. Low physical activity scores in a woman with these risk variables increased the cancer risk. High prevalence of obesity and type 2 diabetes mellitus are seen in patients of PCOS, which are counted as high-risk factors for endometrial cancer as well [30]. Hyperandrogenemia and higher metabolic risk are linked to increased adiposity, especially abdominal adiposity [31]. Additionally, obesity may cluster in PCOS families, and the connection between PCOS and obesity may be exacerbated by referral bias to specialized clinics [32].

When the development of extra weight happens during adolescence rather than during infancy, menstrual difficulties are frequently experienced. Menstrual irregularities and prolonged oligo- or anovulation are more frequent in adolescent overweight and obese patients with PCOS than in normal-weight patients [33]. When given pharmaceuticals to induce ovulation (e.g., gonadotropins, or pulsatile gonadotropin-releasing hormone (GnRH), clomiphene citrate), overweight PCOS patients showed muted responses and reduced pregnancy rates [34,35].

Research on depression that uses various patient populations and techniques of identification shows that women with PCOS have a higher prevalence of depression. Studies employing psychiatric interviews, case-control studies, and community and clinic-based data show that patients suffering from PCOS had a greater prevalence of anxiety and panic disorders [36,37]. Hyperandrogenism, a characteristic of PCOS, and obesity, which is common in PCOS, are considered to contribute to the high prevalence of obstructive sleep apnea (OSA), albeit these factors do not entirely explain the data [38].

Steatosis, liver cell destruction, and inflammation coexist with steatosis in nonalcoholic steatohepatitis (NASH) a subtype of nonalcoholic fatty liver disease (NAFLD). The most common association between insulin resistance and associated phenotypic manifestations is primary NAFLD/NASH. Serum indicators of liver damage may be used to assess women with certain metabolic risk factors, PCOS, and/or insulin resistance [39]. Women with PCOS are more prone to suffer from impaired glucose tolerance (IGT) and type 2 diabetes mellitus as adolescents and adults. A PCOS diagnosis increases the incidence of type 2 diabetes mellitus by five to ten times [40,41]. Additionally, numerous investigations have demonstrated that glucose tolerance declines over time [41]. Numerous scientific organizations advise routine patient screening to identify early alterations in glucose tolerance because of the significant risk of IGT and type 2 diabetes mellitus in PCOS, although a screening interval has not been determined [42,43].

Management

Lifestyle modification: Patients with PCOS are typically recommended to reduce weight as doing so safely can be accomplished through a combination of healthy, balanced food, and frequent physical activity. Because more than half of those with PCOS are overweight or obese [44,45]. Patients with PCOS are overweight, have high blood cholesterol, and have hormonal irregularities. It is essential to realize that physical exercise will never be sufficient for reducing weight on its own. A good diet is more crucial than everything else. Protein and fiber should comprise one gram per kilogram of body weight of a healthy diet. The fact that one must attain a deficit of 30% calories, or 500-750 kilocalories per day, should be highlighted [46]. Research indicates that a loss of 5% of body weight can help in the reestablishment of regular menses and increase the response toward drugs used for reproduction and ovulation [46].

Anovulation/oligo-ovulation: Induction of ovulation is the foundation of medical care for PCOS patients who are facing fertility issues but want to conceive as 70% of PCOS patients suffer difficult ovulation [46]. An example of a selective estrogen receptor modulator (SERM) is clomiphene citrate (CC) [47]. This drug is usually given for five days between the second and fifth day of the menstrual cycle, with a starting dosage of 50 milligrams per day, and can be progressively increased to 150 milligrams per day. Another drug, metformin, can also be used by CC-resistant PCOS women (conditional suggestions supported by evidence, mediocre data). About 30% of successful pregnancies are caused by clomid, while the stillbirth or miscarriage rate for these pregnancies is 20%. Enlargement of the ovary, hyperstimulation syndrome, numerous pregnancies, hot flashes, acidity, bloating, and exhaustion are a few of the side effects [48]. Androgens are converted to estrogen via aromatase [47]. PCOS patients who are ovulating are treated with gonadotropins. If these ovulation-stimulating first-line medications do not work, second-line drugs such as SERM can be given [48].

Insulin resistance and metabolic syndrome: Individuals with PCOS have abnormal insulin secretion and function. There is a strong correlation between ovarian hyperandrogenism and hyperinsulinemia in patients with PCOS. According to studies, hyperinsulinemia stimulates the synthesis of testosterone and androstenedione in the ovarian stroma and thecal cells, which in turn leads to ovarian hyperandrogenism [14,24]. These findings could contribute to the understanding of the robust correlation observed between insulin resistance and polycystic ovary syndrome [48]. High dosages of insulin can harm the ovaries as it affects ovarian function and causes a delay in follicular growth and multiple cysts in the ovary, which is a hallmark of PCOS. Resistance to insulin has long been related to acanthosis nigricans. It is hypothesized that factors that promote the development of dermal fibroblasts and epidermal keratinocytes cause acanthosis nigricans [13,49]. High insulin concentrations can promote the growth of keratinocytes and fibroblasts by binding with great affinity to insulin-like growth factor 1 (IGF-1) receptors. Therefore, for the management of resistance to insulin in PCOS patients, pharmacological modalities and changes in the manner of living are crucial [49].

Type 2 diabetes has long been treated with the safe and effective drug metformin biguanide, which is also one of the most often used for the medication of PCOS. By reducing hepatic glucose synthesis, increasing glucose absorption, and lowering hepatic glucose generation, metformin increases insulin sensitivity in peripheral tissues. Abdominal distension, nausea, vomiting, and diarrhea are a few of the side effects of metformin [47]. Type 2 diabetes or prediabetes are more likely to develop in patients with PCOS [48]. Metformin cures dyslipidemia by lowering high levels of insulin in the blood or free fatty metabolism by the liver [48]. With the initial dose of 500-850 mg daily, metformin is usually prescribed to women with PCOS, and if they show good tolerance to the drug, it can be titrated up to 2,000 mg daily. For those who show intolerance to metformin and experience related adverse effects, different therapy options for PCOS-affected women who are intolerant to the drug should be investigated. An analog of the glucagon-like peptide-1 (GLP-1) receptor increases the release of glucose-dependent insulin, particularly after meals [48].

Hirsutism and acne: Antiandrogen antigens such as spironolactone, flutamide, and finasteride help PCOS sufferers with their hirsutism and acne issues. The effects of flutamide 250 milligrams, spironolactone 100 milligrams, and finasteride 5 milligrams were studied for six months in 40 hirsute women, and results were observed [49]. These antigens were proven to be advantageous for people with increased lipid levels, which is usually observed in women with PCOS. The most popular antiandrogen is spironolactone, which is given in doses of 25-100 milligrams twice a day, which is safe, readily available, and inexpensive [49].

Irregular menstrual cycle: The main mechanism of action of oral contraceptive pills (OCP) is the regulation of the menstrual cycle. These medications also lower testosterone levels, which reduces hirsutism, acne, and hirsutism. The most typical OCP used to treat PCOS-related hirsutism and acne are estrogen and progestogen combinations. Because of the higher potential of thromboembolic impacts, reliance on drugs such as estrogen and synthetic progesterone ought to be reduced and they should not be used as first-line OCP medicines [50].

Infertility: Patients with PCOS who are unable to conceive, with an absence of menstrual bleeding, or with abnormal uterine bleeding should be managed with depot medroxyprogesterone acetate (DMPA). Additionally, medroxyprogesterone acetate (MPA) enhances PCOS patients' lipid profiles and insulin sensitivity [51]. If pharmaceutical ovulation induction drugs fail to treat infertility, assisted reproductive technologies (ART) play an important role. Mainly in vitro fertilization (IVF), intracytoplasmic sperm injection (ICSI), and in vitro manipulation (IVM) are tried [9,51]. Metformin is used as adjuvant therapy for infertility to prevent ovarian hyperstimulation syndrome for patients of PCOS undergoing IVF [2]. 

Obesity: A major indicator of cardiovascular risk is dyslipidemia, which is specified by decreased levels of high-density lipoprotein-cholesterol, high levels of triglyceride, and low-density lipoprotein cholesterol. Statins have been proven to have a crucial role in reducing weight in women with PCOS. It is a medication that stops the production of cholesterol [32,33]. Statins include drugs such as pravastatin, simvastatin, fluvastatin, rosuvastatin, atorvastatin, and rosuvastatin. Treatment with atorvastatin reduced the oxidative stress marker malondialdehyde (MDA) in the blood of obese women with PCOS. Additionally, in this group of PCOS women, atorvastatin reduces androstenedione and dehydroepiandrosterone sulfate levels (DHEAS) [32,49].

By blocking the disintegration of neutral fat in the organs such as the stomach and pancreas, the lipase inhibitor orlistat decreases the absorption of dietary fat. Additionally, orlistat reduces insulin resistance, testosterone levels, and total body fat. Additionally, orlistat lowers blood pressure, and as it aids in weight loss, it may assist those in this high-risk group to avoid type 2 diabetes [52]. Sibutramine, an appetite suppressant, is used to treat obesity together with lifestyle modifications. On the PCOS metabolic component, naltrexone/bupropion may provide clinically substantial weight-loss advantages. Additionally, it can benefit patients who have comorbid diseases such as hypertension, dyslipidemia, preeclampsia, pregnancy-induced diabetes, type 2 diabetes, and large-for-gestational-age children [53]. According to studies, a lack of vitamin D was related to markedly lower chances of having a live birth, pregnancy, and ovulation in women who have been receiving ovarian stimulants for the treatment of infertility [54]. Vitamin D supplements may be helpful for people with metabolic problems, polycystic ovarian syndrome, and ovulation dysfunction [55]. Table 1 provides a summary of all the articles used in the above review article.

**Table 1:** Summary of all articles mentioned in this review article PCOS: polycystic ovarian syndrome; IR: insulin resistance; BMI: body mass index; OSA: obstructive sleep apnea; EDS: excessive daytime sleepiness; NAFLD: nonalcoholic fatty liver disease; OGTT: oral glucose tolerance test; CVD: cardiovascular disease

Serial no.	Authors’ name	Conclusion
1	Deans, 2019 [1]	The majority population of reproductive age group women worldwide suffer from polycystic ovarian syndrome (PCOS)
2	Witchel et al., 2019 [2]	High levels of androgen hormone, impaired insulin sensitivity, and oversized and malfunctioning ovaries are the features associated with PCOS
3	Ndefo et al., 2013 [3]	One out of ten women struggle with PCOS and related issues
4	Bednarska et al., 2017 [4]	Clinical features associated are irregular menstrual cycle, excess facial hair growth, gain of weight, acne, and infertility
5	Ganie et al., 2019 [5]	IR, epigenetics, environmental factors, hyperandrogenism, and genetics are the features associated with PCOS
6	Glueck et al., 2019 [6]	Consequences such as type 2 diabetes mellitus cardiovascular diseases, metabolic syndrome, anxiety, and depression are seen with PCOS
7	Damone et al., 2019 [7]	Anxiety, perceived stress, and depression are higher in women with PCOS
8	Azziz et al., 2006 [8]	Detection of PCOS in its early stages is important for managing its chronic metabolic and reproductive health effects effectively
9	Legro et al., 2013 [9]	Criteria used for PCOS diagnosis are the National Institutes of Health criteria, Rotterdam criteria, androgen excess PCOS society criteria
10	Martin et al., 2008 [10]	Hyperandrogenism, oligo- or anovulation and multiple cysts in ovaries are the frontmost features of PCOS
11	Azziz et al., 2009 [11]	Physical examination includes alopecia, acne, acanthosis nigricans, skin tags, and terminal hair development
12	Semple et al., 2016 [12]	Women may develop male pattern baldness, gain muscular mass, deepen their voice, or develop clitoromegaly in PCOS
13	Sari et al., 2010 [13]	Features such as acanthosis nigricans and skin tags are usually seen in overweight, insulin-resistant women suffering from PCOS
14	Carmina et al., 2006 [14]	In a comprehensive investigation, PCOS was identified in 72.1% of the 950 participants
15	Hatch et al., 1981 [15]	The modified Ferriman-Gallwey score is used to evaluate hirsutism
16	Arias-Santiago et al., 2010 [16]	This study shows the relationship between metabolic syndrome, insulin resistance, and androgenic alopecia
17	Matilainen et al., 2003 [17]	Insulin resistance indicators are associated with a markedly higher incidence of female androgenetic alopecia
18	Ekmekci et al., 2007 [18]	Androgenic alopecia and acne are poor indicators of hyperandrogenism in PCOS
19	Stein et al., 1935 [19]	The primary PCOS symptom is infertility
20	Goldzieher et al., 1963 [20]	Higher population PCOS patients have dominant features of long-term absence of ovulation and hirsutism
21	Balen et al., 1995 [21]	About 50% of PCOS-affected women have primary infertility, and 25% have secondary infertility
22	Hull, 1987 [22]	About 70-90% of ovarian abnormalities seen in PCOS-affected women
23	Trounson et al., 1994 [23]	A novel approach for treating women with PCOS-related infertility could be the recovery of immature oocyte
24	Apparao et al., 2002 [24]	Implantation is prevented by decreased oocyte competence and endometrial alterations
25	Bolúmar et al., 2000 [25]	Subfertility and delayed conception have also been linked to PCOS
26	Godfrey et al., 2000 [26]	Fetal programming of adult illnesses between intrauterine growth restriction and higher risks of type 2 diabetes, ischemic heart disease and hypertension
27	Sir-Petermann et al., 2005 [27]	Some girls who are born tiny for gestational age run the risk of experiencing early adrenarche, IR, or PCOS
28	Diamanti-Kandarakis et al., 2008 [28]	Postpartum obesity and fast weight gain are aggravating factors for developing metabolic abnormalities and PCOS symptoms
29	Dahlgren et al., 1991 [29]	Hirsutism, oligomenorrhea and endometrial cancer in young women increase the likelihood of infertility and nulliparity
30	Folsom et al., 1989 [30]	A high prevalence of obesity and type 2 diabetes mellitus is seen in patients with PCOS, which is a higher risk for the development of endometrial cancer
31	Rosenzweig et al., 2008 [31]	Hyperandrogenia and metabolic risk are linked to abdominal obesity
32	Ezeh et al., 2013 [32]	A PCOS patient is identified by unselected screening or from a referral group has a major impact on the phenotype of PCOS, which includes the racial/ethnic mix, severity of presentation, and rate of obesity
33	Gambineri et al., 2002 [33]	Compared to PCOS women of normal weight, obese women experience more severe hyperandrogenism and associated clinical characteristics (e.g., hirsutism, irregular menstruation, and anovulation)
34	Rausch et al., 2009 [34]	Hirsutism, age, and length of attempted conception are factors that can be used to determine the likelihood of live birth in PCOS women following ovulation induction
35	Kerchner et al., 2009 [35]	Mood disorders are a substantial risk factor for women with PCOS
36	Månsson et al., 2008 [36]	Reduced quality of life and self-rated mental symptoms are linked to PCOS
37	Jedel et al., 2010 [37]	Women with PCOS were identified by several anxiety symptoms from a control group matched for BMI
38	Vgontzas et al., 2001 [38]	Separate from obesity, obstructive sleep apnea (OSA) and excessive daytime sleepiness (EDS) are highly correlated with insulin resistance and hypercytokinemia
39	Kauffman et al., 2010 [39]	The most notable characteristic of NAFLD-exacerbating PCOS was insulin resistance
40	Legro et al., 1991 [40]	A considerable proportion of PCOS women with diabetes were not identified by post-challenge glucose readings using the American Diabetes Association's diabetes diagnostic criteria
41	Ehrmann et al., 1999 [41]	Periodically, women with PCOS should undergo an OGTT and need to be closely watched for any decline in their glucose tolerance
42	American Association of Clinical Endocrinologists Polycystic Ovary Syndrome Writing Committee, 2005 [42]	The impact of sleep issues, obesity, and PCOS's neuropsychological features, as well as the pertinent pathogenetic elements of cardiovascular risk factors
43	Wild et al., 2010 [43]	Lifestyle management is recommended for primary CVD prevention, targeting low-density and non-high-density lipoprotein cholesterol and adding insulin-sensitizing and other drugs if dyslipidemia or other risk factors persist
44	Wang et al., 2017 [44]	Weight reduction, healthy and balanced food with frequent physical activity should be taken as first-line management for PCOS management
45	Dai et al., 2021 [45]	More than half of those with PCOS are overweight or obese
46	Day et al., 2018 [46]	Additionally, the data offer the first genetic proof of a male PCOS phenotype and a link to depression
47	Trent et al., 2020 [47]	Hyperandrogenism, dysfunctional ovaries, and irregular menses are the top clinical features for the diagnosis of PCOS
48	Palomba et al., 2014 [48]	When gonadotropins are used to induce ovulation in PCOS patients, the rate of live births and pregnancies rises when metformin is administered
49	Jia et al., 2021 [49]	PCOS patients have aberrant insulin function and secretion
50	de Melo et al., 2017 [50]	The first choice of PCOS therapy options are combined hormonal contraception
51	Tanbo et al., 2018 [51]	Frequent exercise can lead to ovulation on its own and increase the chances of an ovulation induction successfully
52	Panda et al., 2018 [52]	In comparison to metformin, orlistat is a safe and efficient medication for polycystic ovarian syndrome
53	de Leo et al., 2016 [53]	Therapeutic tools for PCOS are metformin, hormonal contraceptives, inositol, and anti-androgen medication
54	Cunha et al., 2021 [54]	Gonadotropins should be used as second-line management for women who have failed first-line oral ovulation inducers
55	GBD 2019 Diseases and Injuries Collaborators, 2020 [55]	Vitamin D supplements are shown to be helpful for people with metabolic problems, PCOS, and ovulation dysfunction

## Conclusions

A complex hormonal, metabolic, and psychological illness, PCOS has a broad range of clinical outcomes such as irregular menstrual cycle, hirsutism, acne, weight gain, and infertility. While its other associated co-comorbidities are metabolic syndrome, infertility, and ischemic heart disease, it is among the most prevalent causes of infertility. Rotterdam criteria are most used to diagnose PCOS (presence of two of the following criteria: androgen excess, ovulatory dysfunction, or polycystic ovaries). Lifestyle modifications should be thought of as a milestone for the management of PCOS. OCPs are used as a mainstay for managing irregular menstrual cycles. Ovulation inducers are used in the treatment of anovulation in PCOS. CC is currently used as first-line management of infertility in PCOS. Metformin is used to treat metabolic and glycemic abnormalities in PCOS patients. Antiandrogens such as spironolactone and flutamide are used in treating hirsutism in PCOS patients. Statins class of drugs are beneficial to reduce weight in women with PCOS.
